# Supplementary material for: A Synthetic Formula Amino Acid Diet Leads to Microbiome Dysbiosis, Reduced Colon Length, Inflammation, and Altered Locomotor Activity in C57BL/6J Mice
Source: Microorganisms. 2023 Nov 3;11(11):2694. doi: 10.3390/microorganisms11112694 (PMC10673175; doi:10.3390/microorganisms11112694)
Supplement: Supplementary file 1 [file microorganisms-11-02694-s001.zip › microorganisms-2641477-supplementary/Mancilla_Supplementary Material 10.23.23 acceptedchanges.pdf]

A Synthetic Formula Amino Acid Diet Leads to Microbiome Dysbiosis, Reduced Colon Length, Inflammation, and Altered Locomotor Activity in C57BL/6J Mice

Viviana J. Mancilla[1], Paige N. Braden-Kuhle[2], Kelly N. Brice[2], Allison E. Mann[1,4], Megan T. Williams[1], Yan Zhang[1], Michael J. Chumley[3], Robert C. Barber[5], Sabrina N. White[1], Gary W. Boehm[2*], Michael S. Allen[1*]

*** Correspondence:** Michael Allen & Gary Boehm
Michael.Allen@unthsc.edu (M.S.A), g.boehm@tcu.edu (G.W.B)

1 Supplementary Figures and Tables

1.1 Supplementary Figures

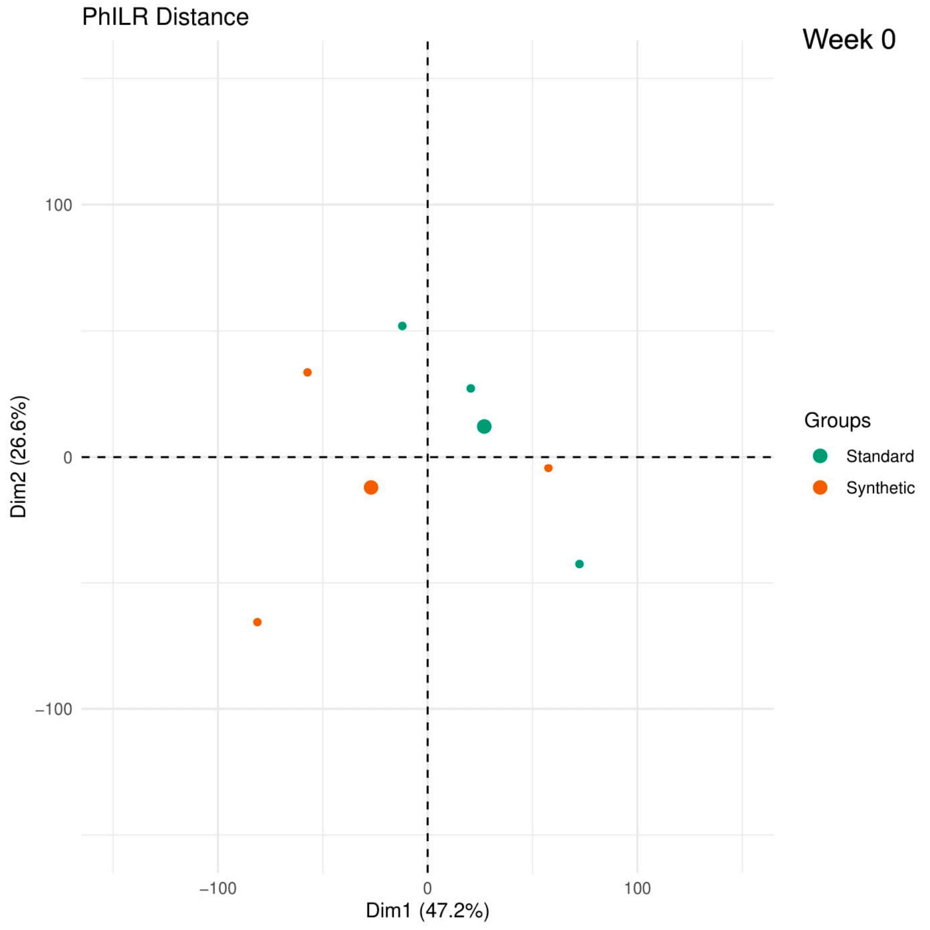

Supplementary Figure S1. Principal component analysis (PCA) of PhILR distances for the gut microbiota of synthetic and standard diet animals prior to diet consumption. Fecal samples collected at weaning. Individual samples are represented by one point; larger circles represent the mean coordinates for each diet group.

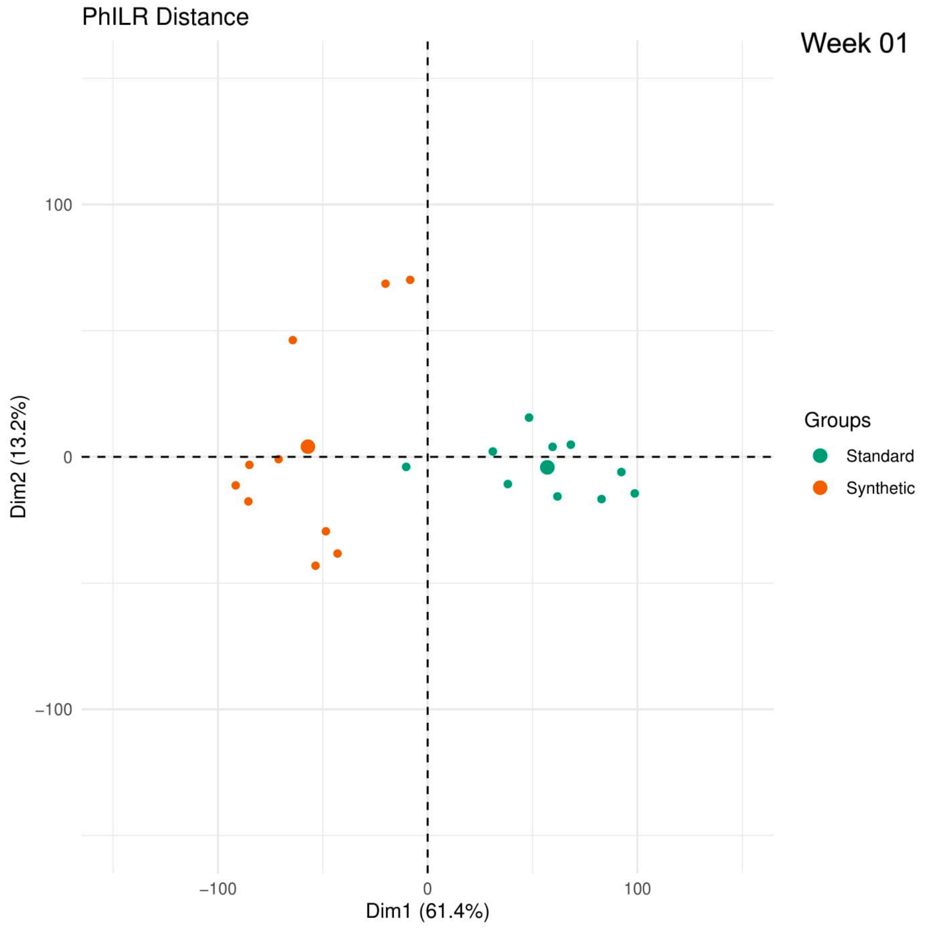

Supplementary Figure S2. Principal component analysis (PCA) of PhILR distances for the gut microbiota of synthetic and standard diet animals after 1 week of consuming the assigned diet. Individual samples are represented by one point; larger circles represent the mean coordinates for each diet group.

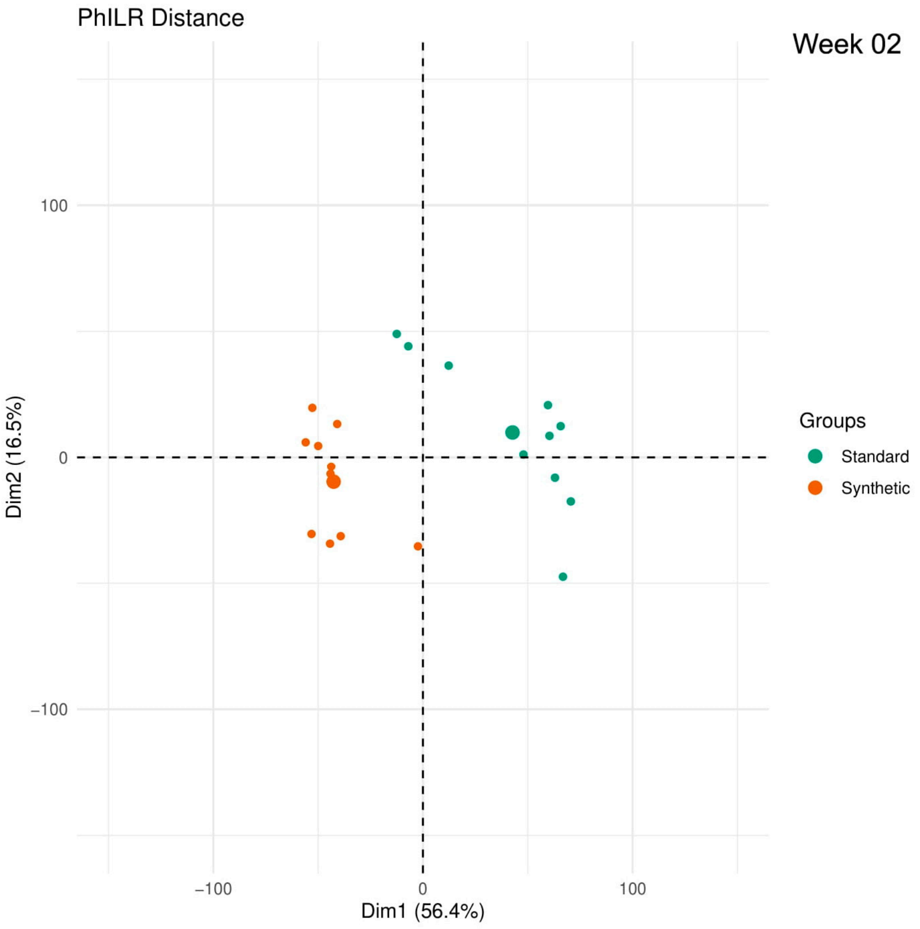

Supplementary Figure S3. Principal component analysis (PCA) of PhILR distances for the gut microbiota of synthetic and standard diet animals after 2 weeks of consuming the assigned diet. Individual samples are represented by one point; larger circles represent the mean coordinates for each diet group.

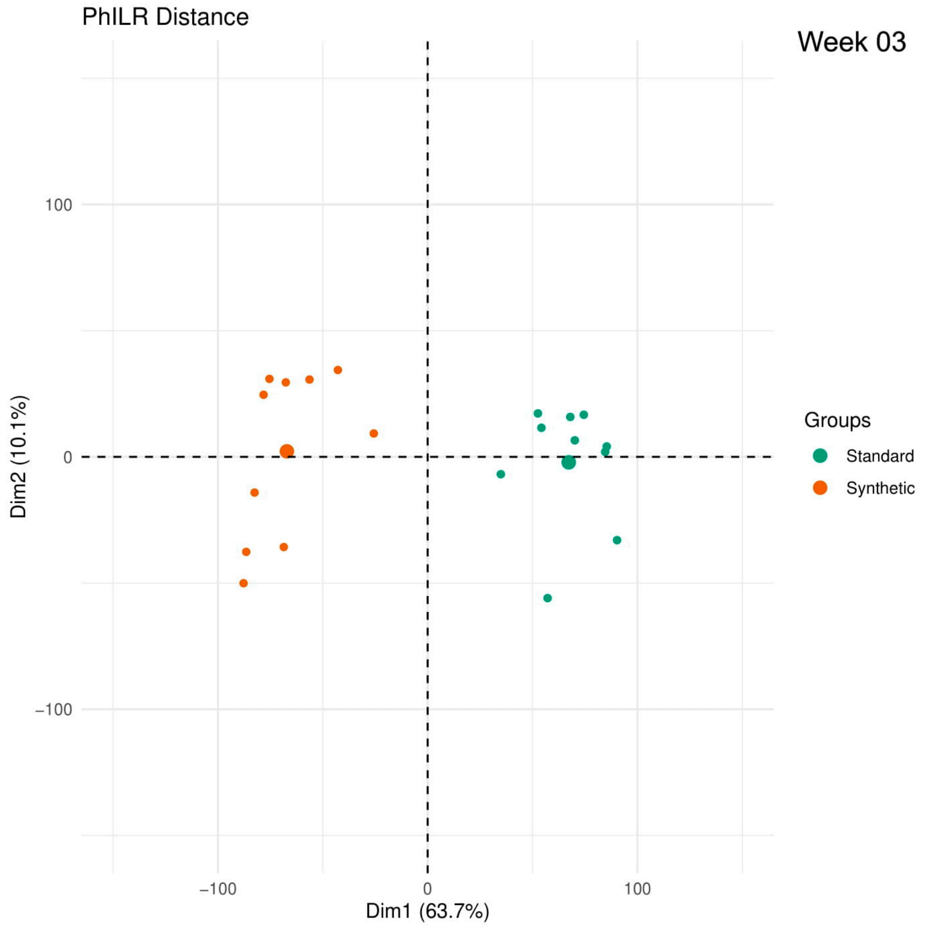

Supplementary Figure S4. Principal component analysis (PCA) of PhILR distances for the gut microbiota of synthetic and standard diet animals after 3 weeks of consuming the assigned diet. Individual samples are represented by one point; larger circles represent the mean coordinates for each diet group.

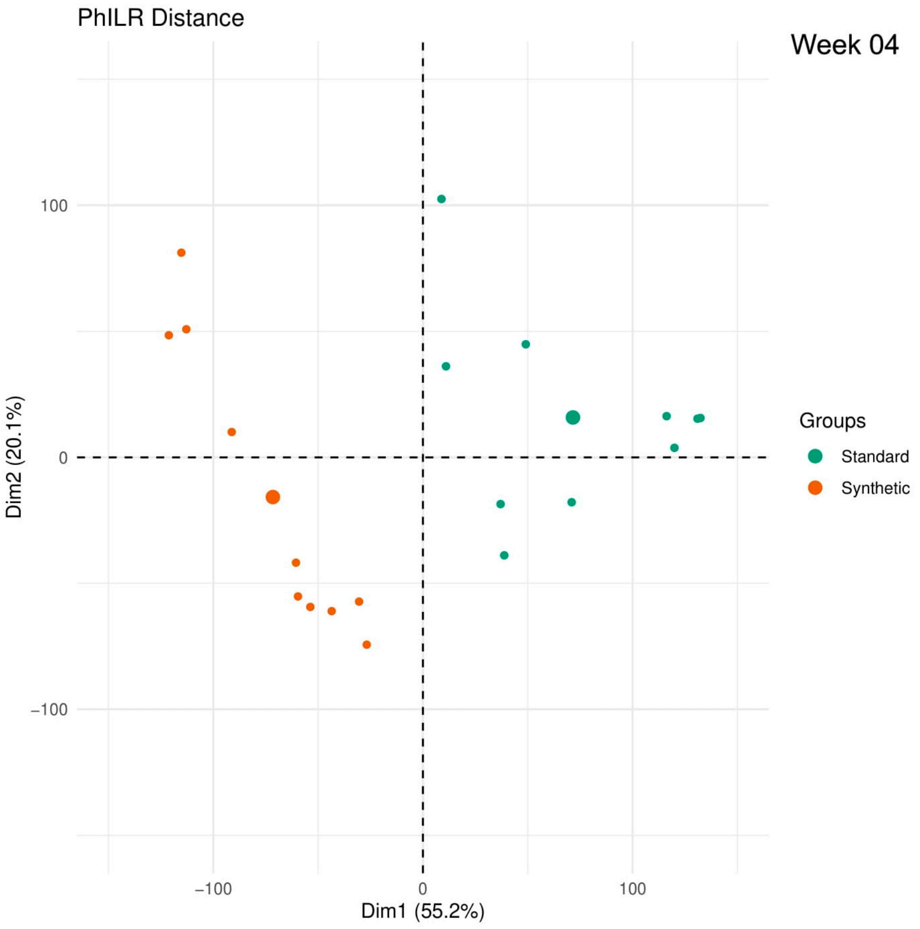

Supplementary Figure S5. Principal component analysis (PCA) of PhILR distances for the gut microbiota of synthetic and standard diet animals after 4 weeks of consuming the assigned diet. Individual samples are represented by one point; larger circles represent the mean coordinates for each diet group.

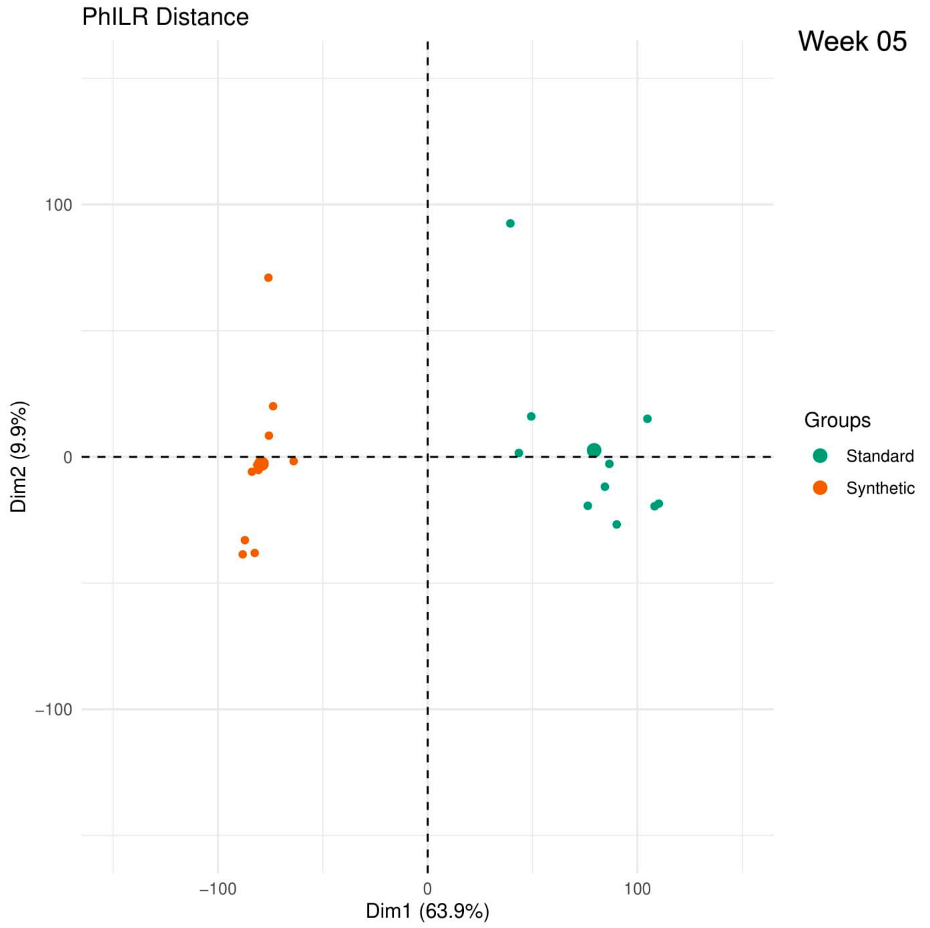

Supplementary Figure S6. Principal component analysis (PCA) of PhILR distances for the gut microbiota of synthetic and standard diet animals after 5 weeks of consuming the assigned diet. Individual samples are represented by one point; larger circles represent the mean coordinates for each diet group.

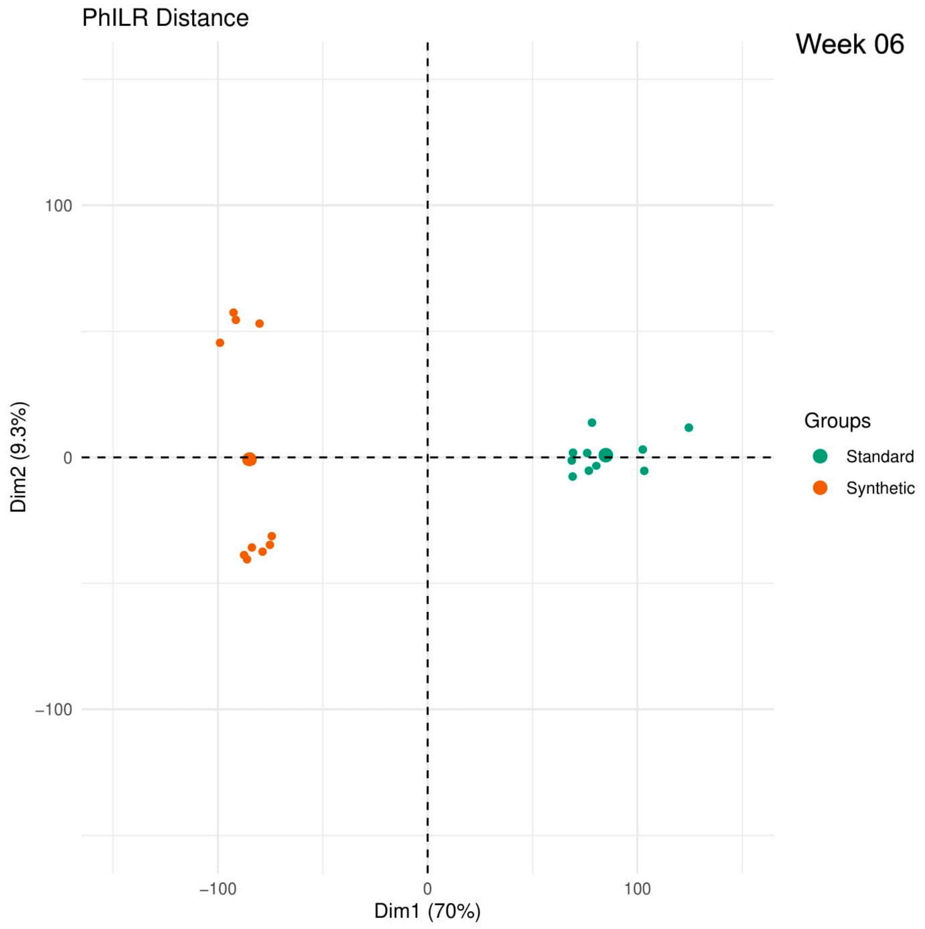

Supplementary Figure S7. Principal component analysis (PCA) of PhILR distances for the gut microbiota of synthetic and standard diet animals after 6 weeks of consuming the assigned diet. Individual samples are represented by one point; larger circles represent the mean coordinates for each diet group.

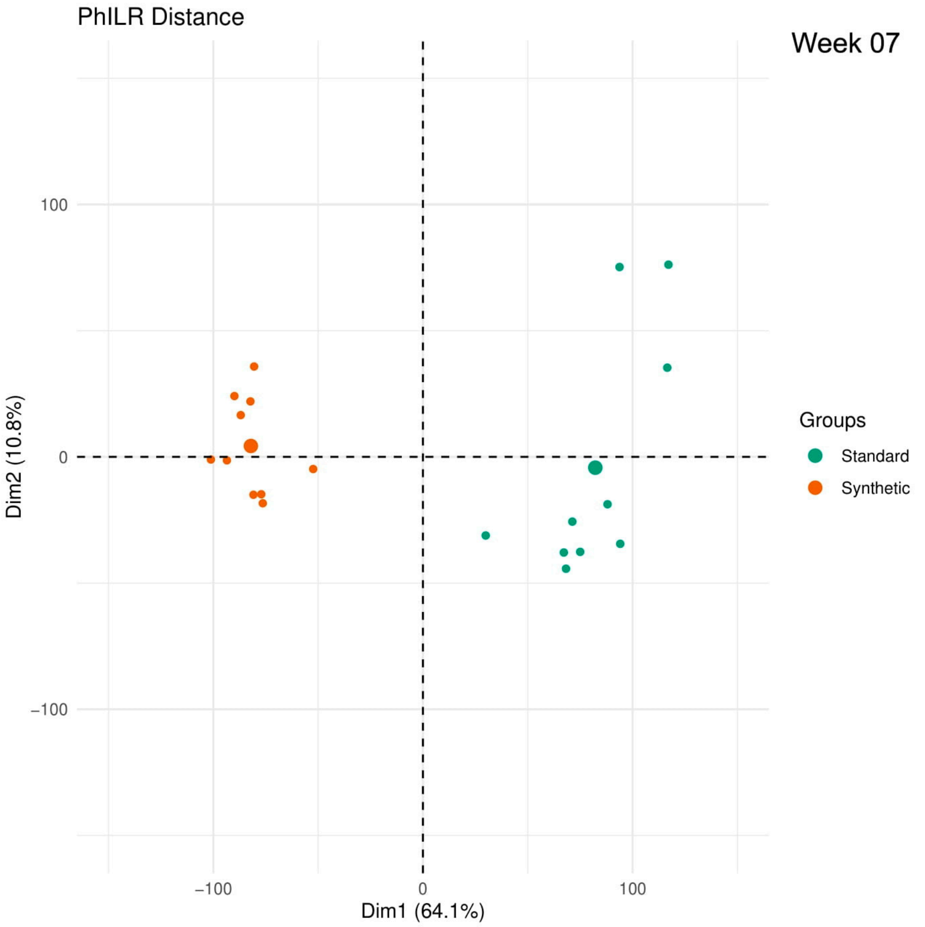

Supplementary Figure S8. Principal component analysis (PCA) of PhILR distances for the gut microbiota of synthetic and standard diet animals after 7 weeks of consuming the assigned diet. Individual samples are represented by one point; larger circles represent the mean coordinates for each diet group.

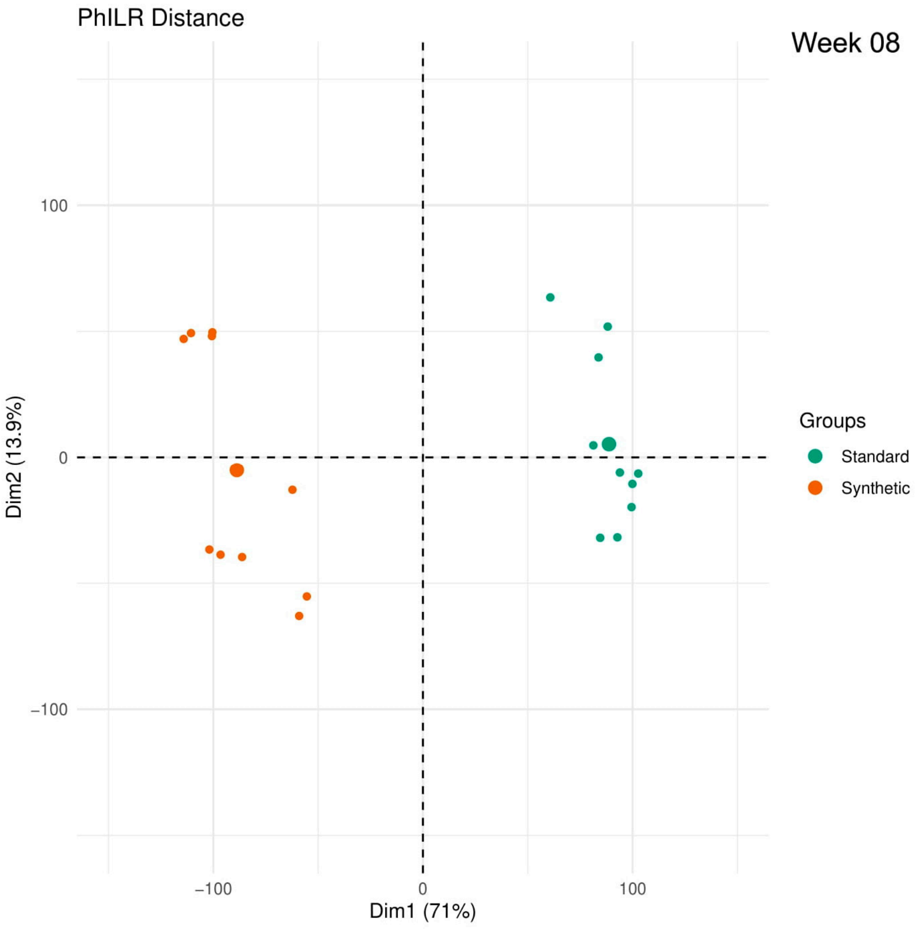

Supplementary Figure S9. Principal component analysis (PCA) of PhILR distances for the gut microbiota of synthetic and standard diet animals after 8 weeks of consuming the assigned diet. Individual samples are represented by one point; larger circles represent the mean coordinates for each diet group.

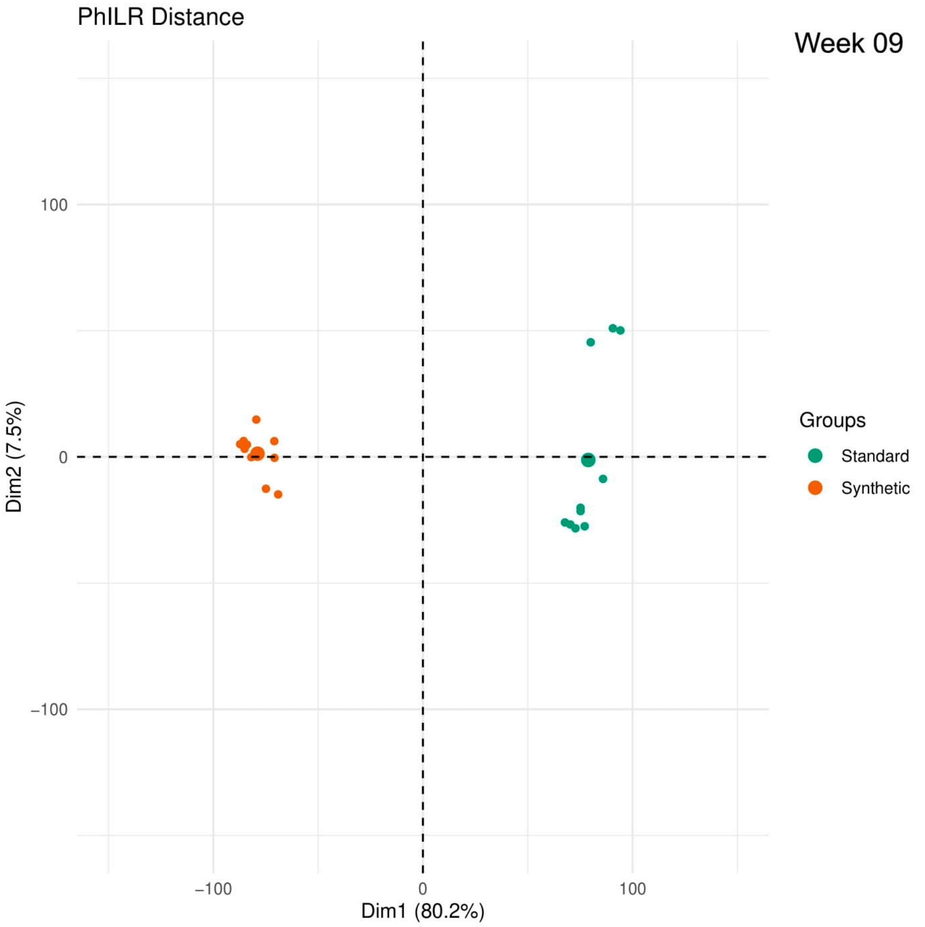

Supplementary Figure S10. Principal component analysis (PCA) of PhILR distances for the gut microbiota of synthetic and standard diet animals after 9 weeks of consuming the assigned diet. Individual samples are represented by one point; larger circles represent the mean coordinates for each diet group.

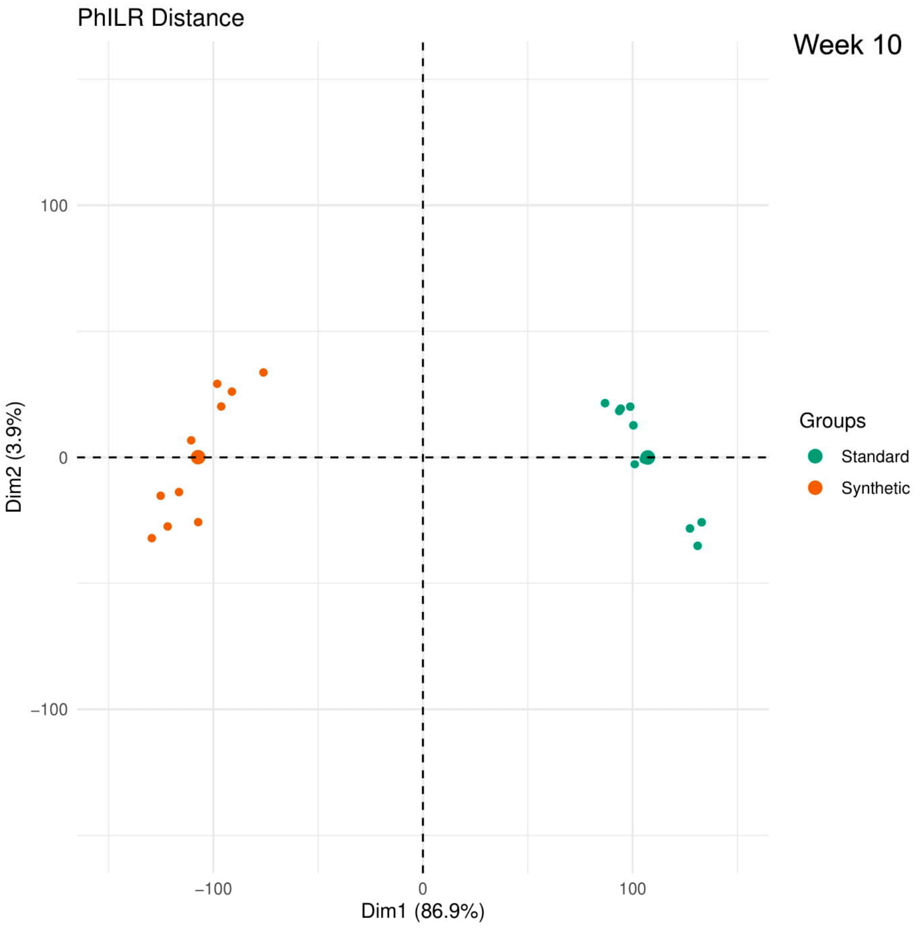

Supplementary Figure S11. Principal component analysis (PCA) of PhILR distances for the gut microbiota of synthetic and standard diet animals after 10 weeks of consuming the assigned diet. Individual samples are represented by one point; larger circles represent the mean coordinates for each diet group.

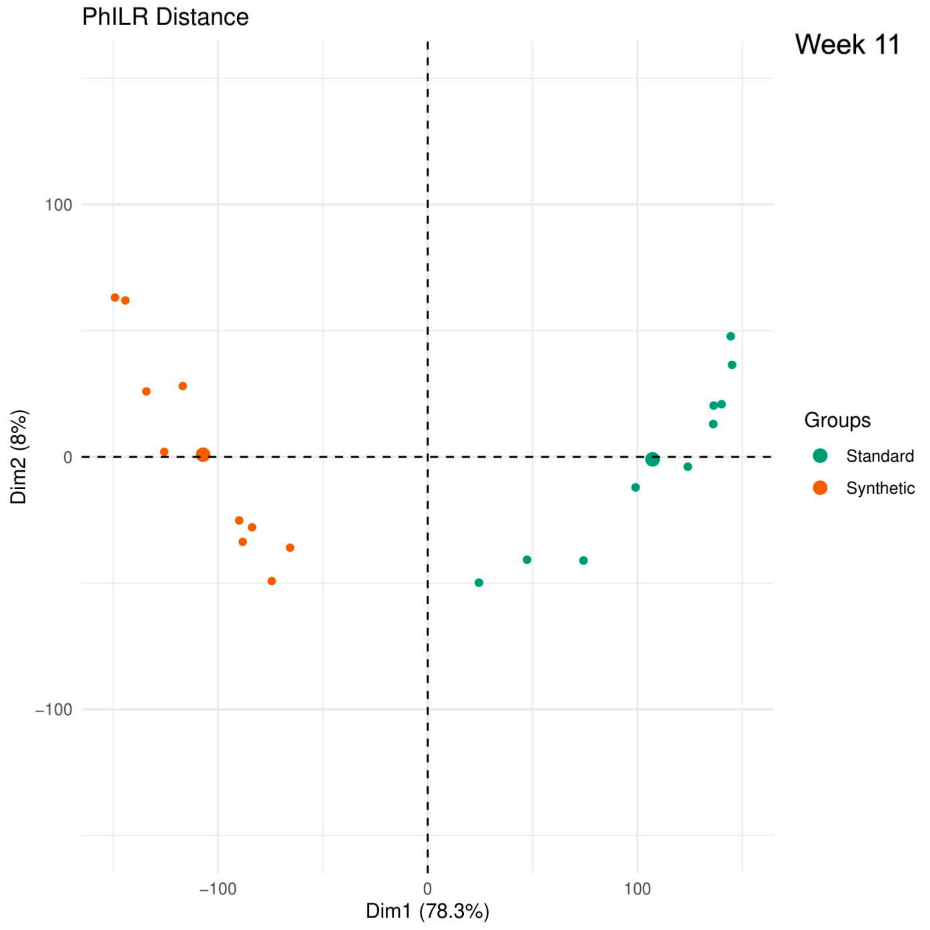

Supplementary Figure S12. Principal component analysis (PCA) of PhILR distances for the gut microbiota of synthetic and standard diet animals after 11 weeks of consuming the assigned diet. Individual samples are represented by one point; larger circles represent the mean coordinates for each diet group.

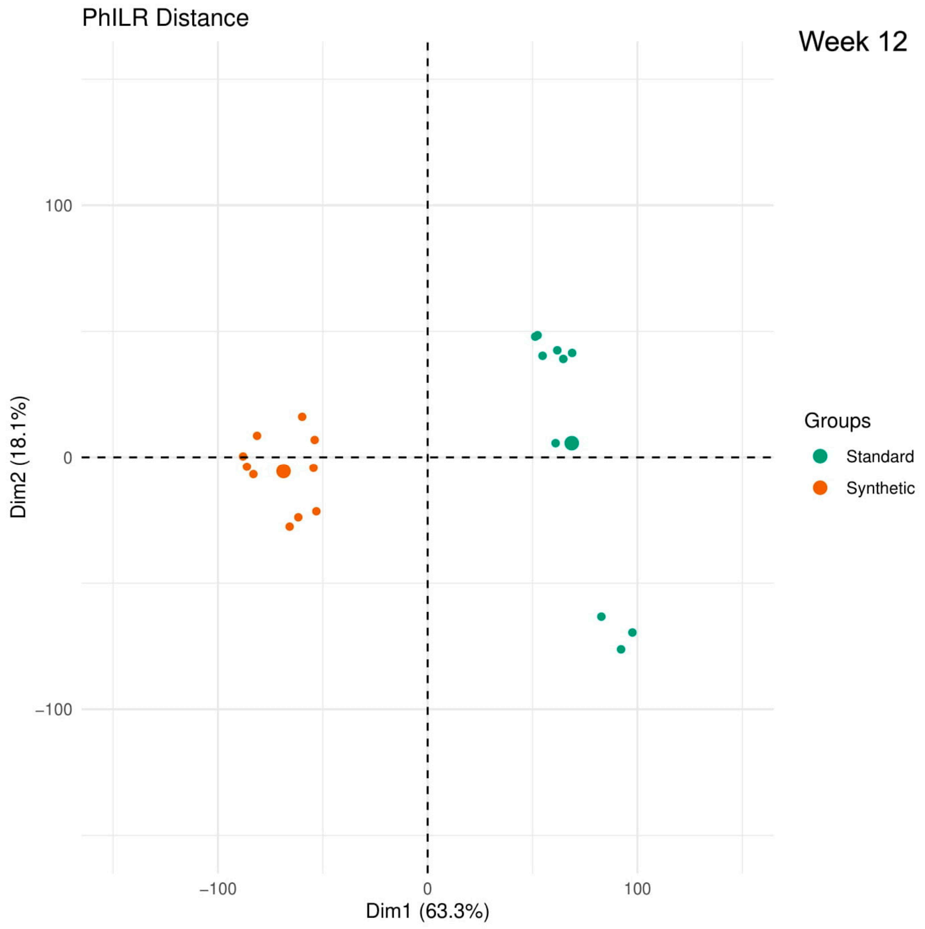

Supplementary Figure S13. Principal component analysis (PCA) of PhILR distances for the gut microbiota of synthetic and standard diet animals after 12 weeks of consuming the assigned diet. Individual samples are represented by one point; larger circles represent the mean coordinates for each diet group.

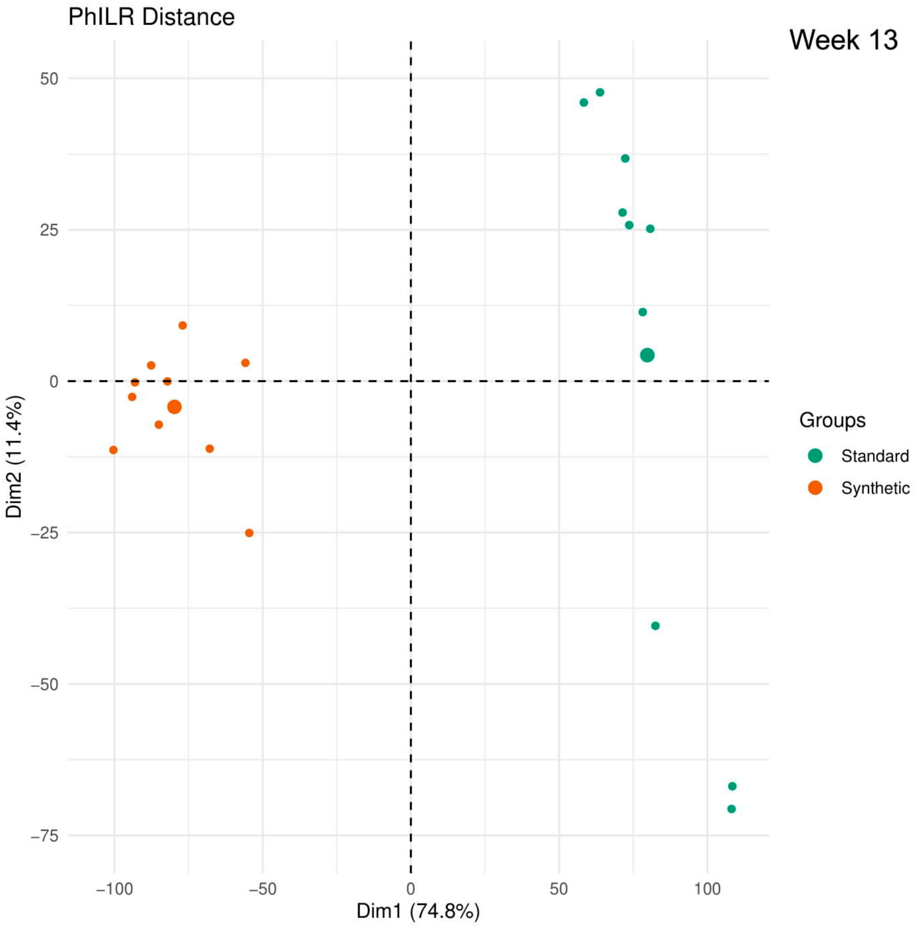

Supplementary Figure S14. Principal component analysis (PCA) of PhILR distances for the gut microbiota of synthetic and standard diet animals after 13 weeks of consuming the assigned diet. Individual samples are represented by one point; larger circles represent the mean coordinates for each diet group.

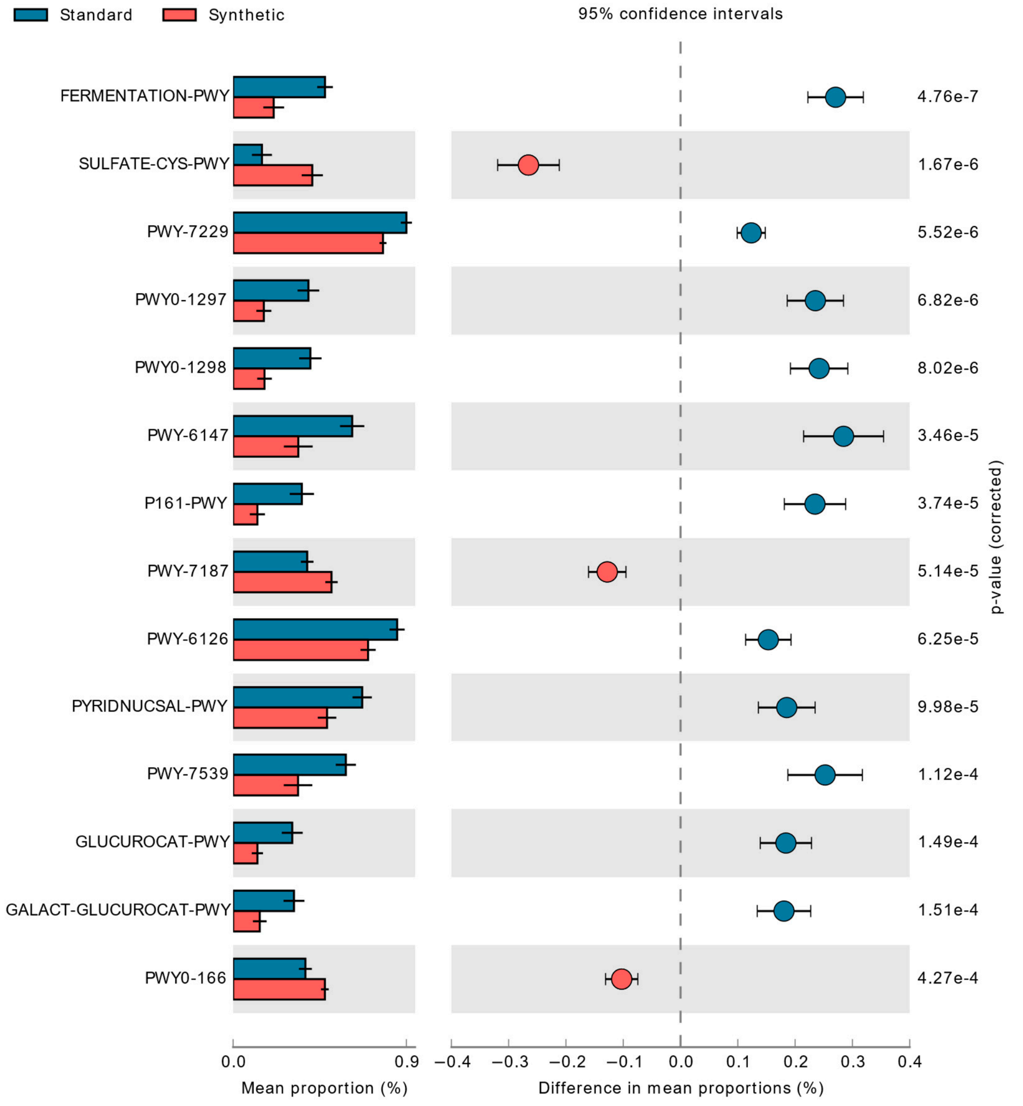

Supplementary Figure S15. PICRUSt2 analysis predicted 14 metabolic pathways may be affected by the consumption of the synthetic formula diet (coral) compared to the standard diet (blue), based on microbiota present in the gut microbiome in mice in each dietary group at week 13. The colored bars represent 95% confidence intervals calculated using Welch's inverted method ($p \leq 0.05$ with Bonferroni corrections for multiple comparisons. The colored circles represent the difference in mean proportions between synthetic diet and standard diet animal samples.

1.2 Supplementary Tables

	**Standard**<br>% by weight	**Synthetic**<br>% by weight
**Protein**	18.2	18.2
**CHO**	55.8	60.3
**Fat**	5.2	5.2
**Crude Fiber**	4.9	5%
**kcal/g**	3.43	3.6

Supplemental Table S1. Nutrient Information

**Standard Diet Ingredients:**
Ground Corn
Wheat Middlings
Dehulled Soybean Meal
Ground Wheat
Fish Meal
Porcine Animal Fat Preserved with BHA and Citric Acid
Dehydrated Alfalfa Meal
Cane Molasses
Ground Oats
Wheat Germ
Calcium Carbonate
Brewers dried Yeast
Salt
Dried Beet Pulp
Corn Gluten Meal
Ground Soy-Bean Hulls
Soybean Oil
Dried Whey
Porcine Meat and Bone Meal

Dicalcium Phosphate
L-Lysine
Sucrose
DL-Methionine
Menadione Dimethylpyrimidinol Bisulfite
Magnesium Oxide
Vitamin A Acetate
Cholecalciferol
Biotin
DL-Alpha Tocopheryl Acetate
Riboflavin Supplement
Folic Acid
Vitamin B-12 Supplement
Nicotinic Acid
Calcium Pantothenate
Pyridoxine Hydrochloride
Manganous Oxide
Zinc Oxide
Ferrous Carbonate
Copper Sulfate

Zinc Sulfate
Calcium Iodate
Cobalt Carbonate
Sodium Selenite

Supplemental Table S2. List of Ingredients in Standard Diet

**Synthetic Diet Ingredients:**
L-Alanine
L-Arginine HCl
L-Asparagine
L-Aspartic Acid
L-Cystine
L-Glutamic Acid
L-Glutamine
Glycine
L-Histidine HCl, monohydrate
L-Isoleucine
L-Leucine
L-Lysine HCl
L-Methionine

L-Phenylalanine
L-Proline
L-Serine
L-Threonine
L-Tryptophan
L-Tyrosine
L-Valine
Taurine
Sucrose
Corn Starch
Maltodextrin
Lard
Soybean Oil
Cellulose
Mineral Mix, AIN-93M-MX (94049)
Calcium Phosphate, monobasic, monohydrate
Vitamin Mix, AIN-93-VX (94047)
Choline Bitartrate
TBHQ, antioxidant

Supplemental Table S3. List of Ingredients in the Synthetic Diet

Diet Condition	% Insoluble Fiber	% Soluble Fiber
**Synthetic Diet**	5%	0%
**Standard Control Diet**	8.58%	1.2%

Supplemental Table S4. Fiber Content in Experimental Diets

Pathway	p-value	In synthetic vs standard	MetaCyc Pathway
FERMENTATION-PWY	4.76E-07	lower	mixed acid fermentation
SULFATE-CYS-PWY	1.67E-06	higher	superpathway of sulfate assimilation and cysteine biosynthesis
PWY-7229	5.52E-06	lower	superpathway of adenosine nucleotides de novo biosynthesis I
PWY0-1297	6.82E-06	lower	superpathway of purine deoxyribonucleosides degradation
PWY0-1298	8.02E-06	lower	superpathway of pyrimidine deoxyribonucleosides degradation
PWY-6147	3.46E-05	lower	6-hydroxymethyl-dihydropterin diphosphate biosynthesis I
P161-PWY	3.74E-05	lower	acetylene degradation (anaerobic)
PWY-7187	5.14E-05	higher	pyrimidine deoxyribonucleotides de novo biosynthesis II

PWY-6126	6.25E-05	lower	superpathway of adenosine nucleotides de novo biosynthesis II
PYRIDNUCSAL-PWY	9.98E-05	lower	NAD salvage pathway I (PNC VI cycle)
PWY-7539	1.12E-04	lower	6-hydroxymethyl-dihydropterin diphosphate biosynthesis III (Chlamydia)
GLUCUROCAT-PWY	1.49E-04	lower	superpathway of β-D-glucuronosides degradation
GALACT-GLUCUROCAT-PWY	1.51E-04	lower	superpathway of hexuronide and hexuronate degradation
PWY0-166	4.27E-04	higher	superpathway of pyrimidine deoxyribonucleotides de novo biosynthesis (E. coli)
GLYOXYLATE-BYPASS	1.32E-03	higher	glyoxylate cycle
PWY-5659	1.41E-03	higher	GDP-mannose biosynthesis
PWY-6385	1.56E-03	lower	peptidoglycan biosynthesis III (mycobacteria)
SO4ASSIM-PWY	2.00E-03	higher	assimilatory sulfate reduction I
PWY-7199	2.12E-03	lower	pyrimidine deoxyribonucleosides salvage
PWY-7197	4.07E-03	higher	pyrimidine deoxyribonucleotide phosphorylation
PWY-7222	5.33E-03	lower	guanosine deoxyribonucleotides de novo biosynthesis II
PWY-7220	5.33E-03	lower	adenosine deoxyribonucleotides de novo biosynthesis II
BIOTIN-BIOSYNTHESIS-PWY	7.11E-03	higher	biotin biosynthesis I

PWY-5971	9.65E-03	higher	palmitate biosynthesis (type II fatty acid synthase)
PWY-5667	0.01	lower	CDP-diacylglycerol biosynthesis I
PWY0-1319	0.01	lower	CDP-diacylglycerol biosynthesis II
PWY0-1586	0.01	lower	peptidoglycan maturation (meso-diaminopimelate containing)
PWY-7392	0.011	higher	taxadiene biosynthesis (engineered)
PWY-7664	0.012	higher	oleate biosynthesis IV (anaerobic)
PWYG-321	0.012	higher	mycolate biosynthesis
PWY-6891	0.013	higher	thiazole component of thiamine diphosphate biosynthesis II
PWY0-862	0.014	higher	(5Z)-dodecenoate biosynthesis I
PWY-6282	0.015	higher	palmitoleate biosynthesis I (from (5Z)-dodec-5-enoate)
PWY-6519	0.015	higher	8-amino-7-oxononanoate biosynthesis I
PWY-5484	0.016	higher	glycolysis II (from fructose 6-phosphate)
PWY-5989	0.017	higher	stearate biosynthesis II (bacteria and plants)
FASYN-INITIAL-PWY	0.018	higher	superpathway of fatty acid biosynthesis initiation (E. coli)
GALACTUROCAT-PWY	0.021	lower	D-galacturonate degradation I
P441-PWY	0.022	lower	superpathway of N-acetylneuraminate degradation
PWY-2942	0.025	lower	L-lysine biosynthesis III

PWY-7456	0.034	higher	β-(1,4)-mannan degradation
POLYAMINSYN3-PWY	0.034	lower	superpathway of polyamine biosynthesis II
HEMESYN2-PWY	0.035	lower	heme b biosynthesis II (oxygen-independent)
PWY-6353	0.042	lower	purine nucleotides degradation II (aerobic)
PWY-7184	0.046	higher	pyrimidine deoxyribonucleotides de novo biosynthesis I
ARG+POLYAMINE-SYN	0.048	lower	superpathway of arginine and polyamine biosynthesis

Supplemental Table S5. PICRUSt2 Results Comparing Data Collected from Mice Fed a Synthetic Diet or the Standard Diet after 13 Weeks post-weaning, $p \leq 0.05$.
