# Supplementary material for: A Synthetic Formula Amino Acid Diet Leads to Microbiome Dysbiosis, Reduced Colon Length, Inflammation, and Altered Locomotor Activity in C57BL/6J Mice
Source: Microorganisms. 2023 Nov 3;11(11):2694. doi: 10.3390/microorganisms11112694 (PMC10673175; doi:10.3390/microorganisms11112694)
Supplement: Supplementary file 1 [file microorganisms-11-02694-s001.zip › microorganisms-2641477-supplementary/philr_pca_fitted_11.pdf]

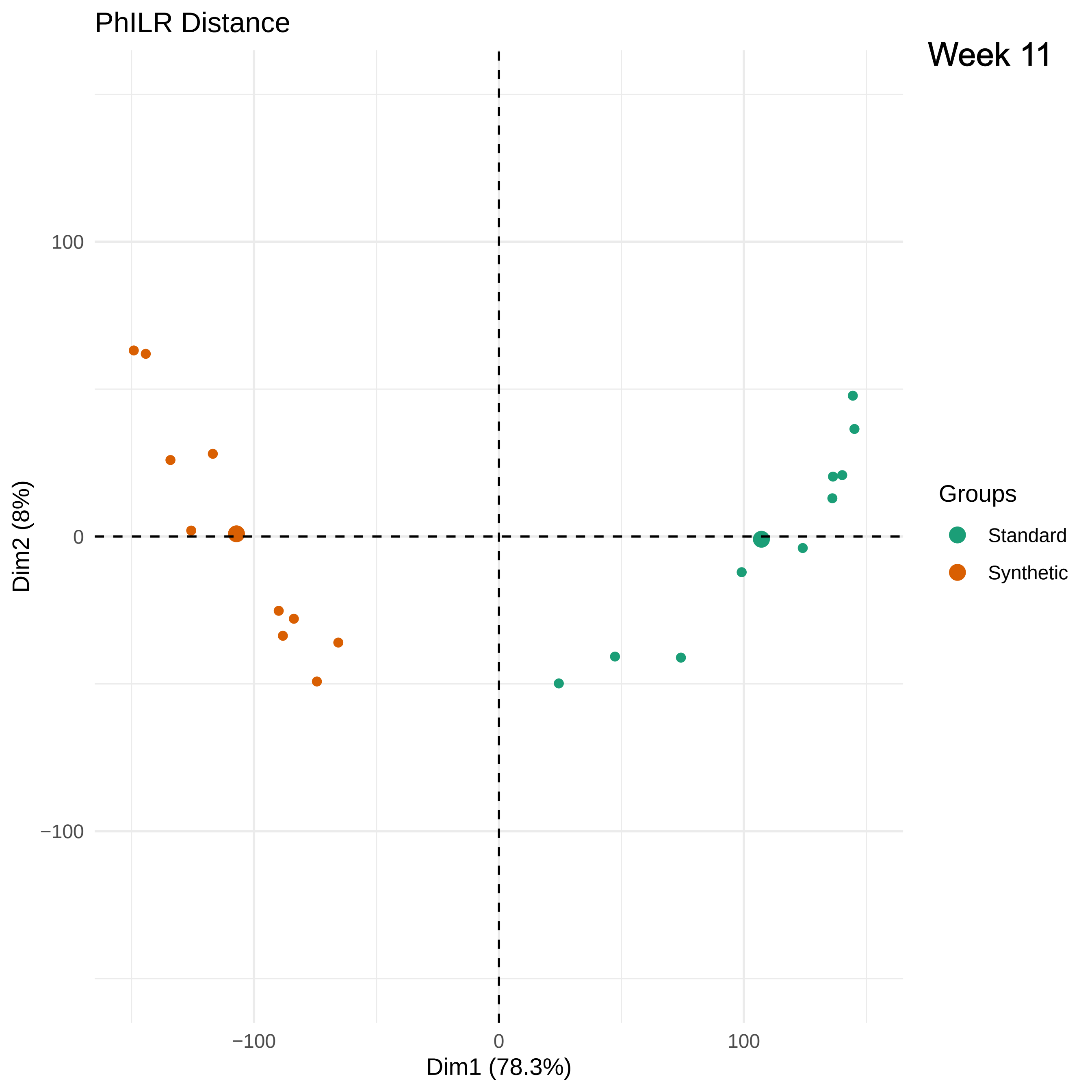
PhILR Distance
Week 11
100
0
−100
Dim2 (8%)
−100
0
100
Dim1 (78.3%)
Groups
Standard
Synthetic
