# Supplementary figures and images for: A Synthetic Formula Amino Acid Diet Leads to Microbiome Dysbiosis, Reduced Colon Length, Inflammation, and Altered Locomotor Activity in C57BL/6J Mice
Source: Microorganisms. 2023 Nov 3;11(11):2694. doi: 10.3390/microorganisms11112694 (PMC10673175; doi:10.3390/microorganisms11112694)

# Week 01

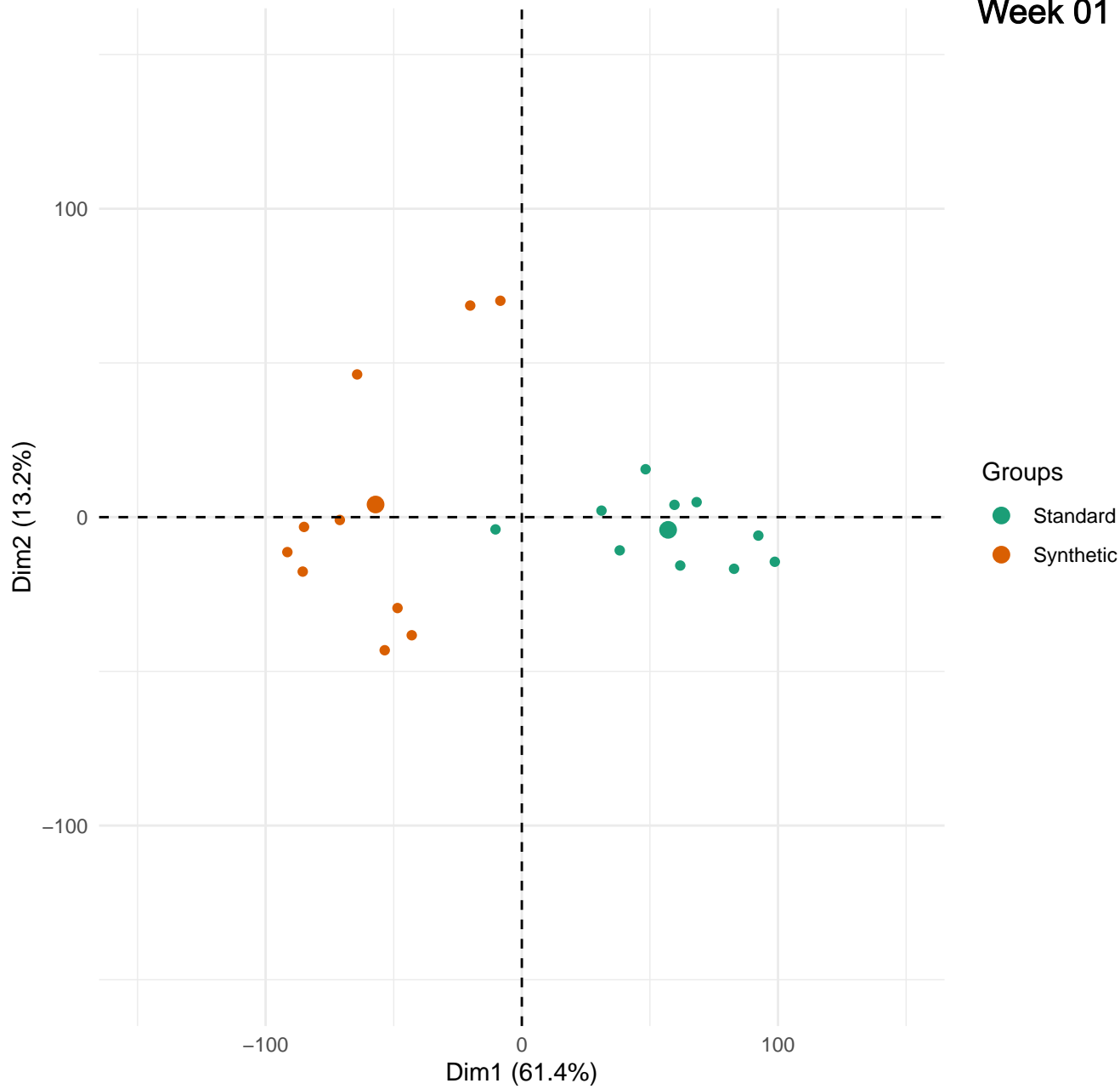

Supplement: Supplementary file 1 [file microorganisms-11-02694-s001.zip › microorganisms-2641477-supplementary/philr_pca_fitted_01.pdf]

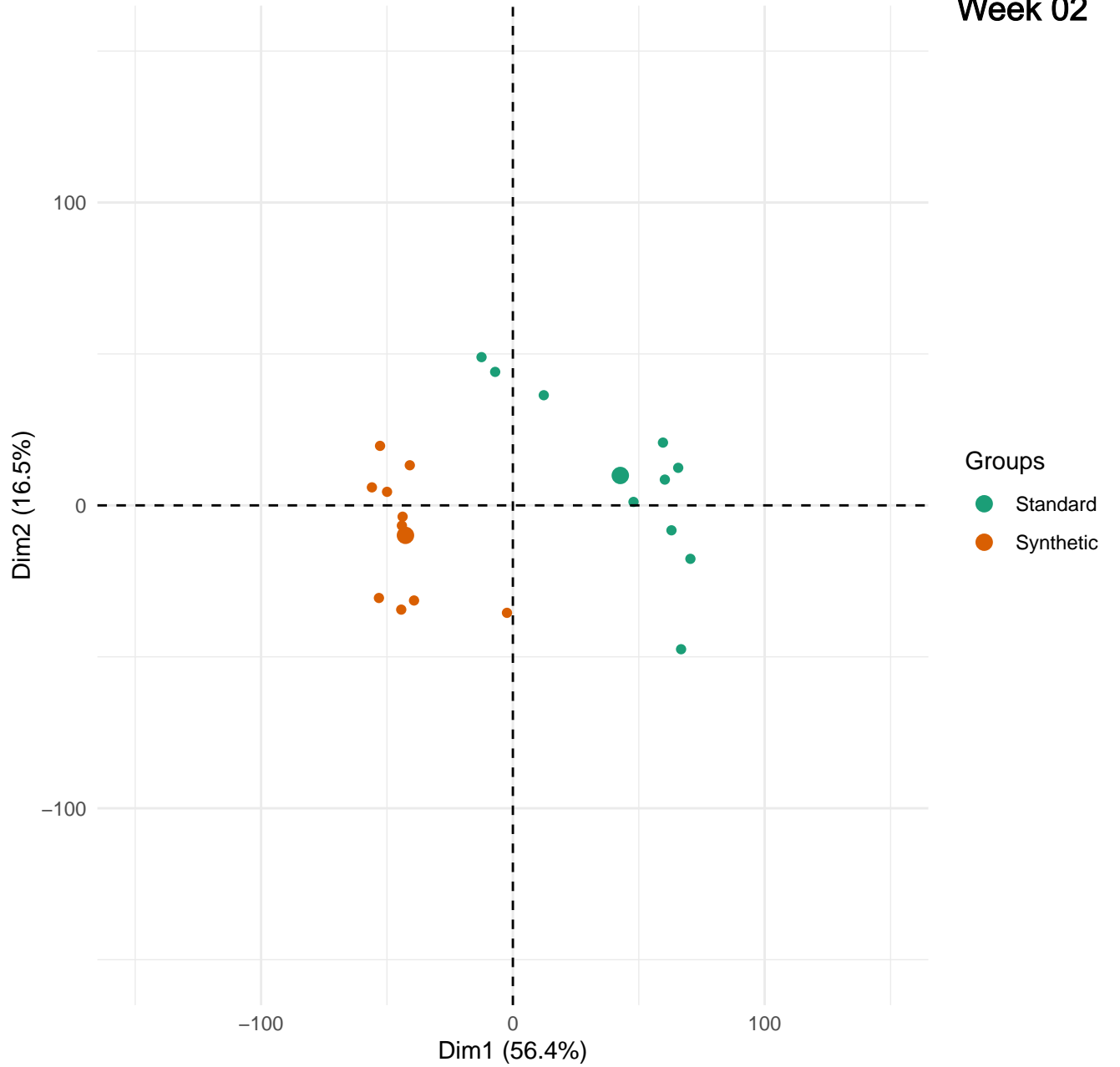

Supplement: Supplementary file 1 [file microorganisms-11-02694-s001.zip › microorganisms-2641477-supplementary/philr_pca_fitted_02.pdf]

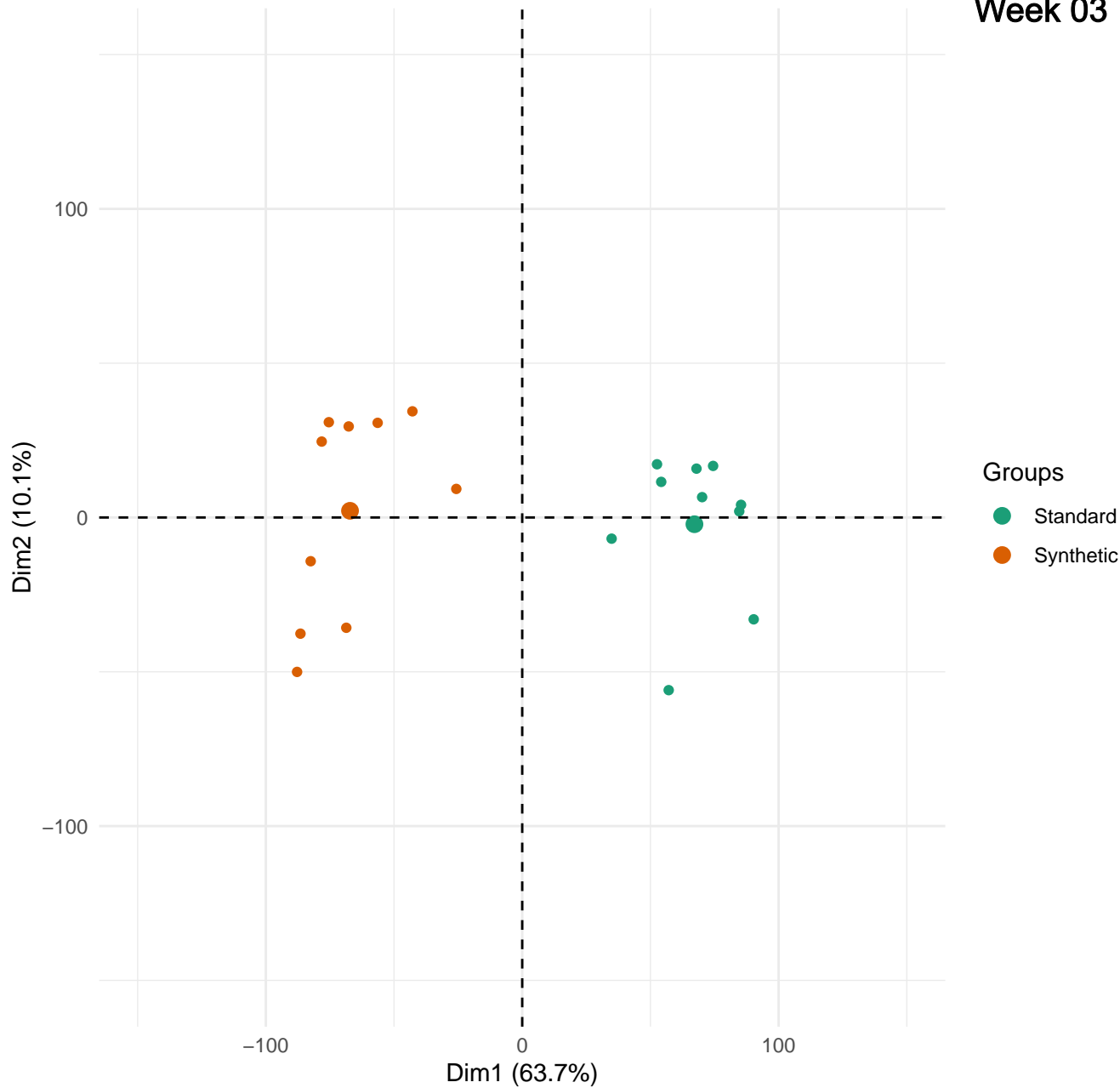

Supplement: Supplementary file 1 [file microorganisms-11-02694-s001.zip › microorganisms-2641477-supplementary/philr_pca_fitted_03.pdf]

Dim2 (20.1%)

Dim1 (55.2%)

Groups

- Standard
- Synthetic

100

0

-100

-100

0

100

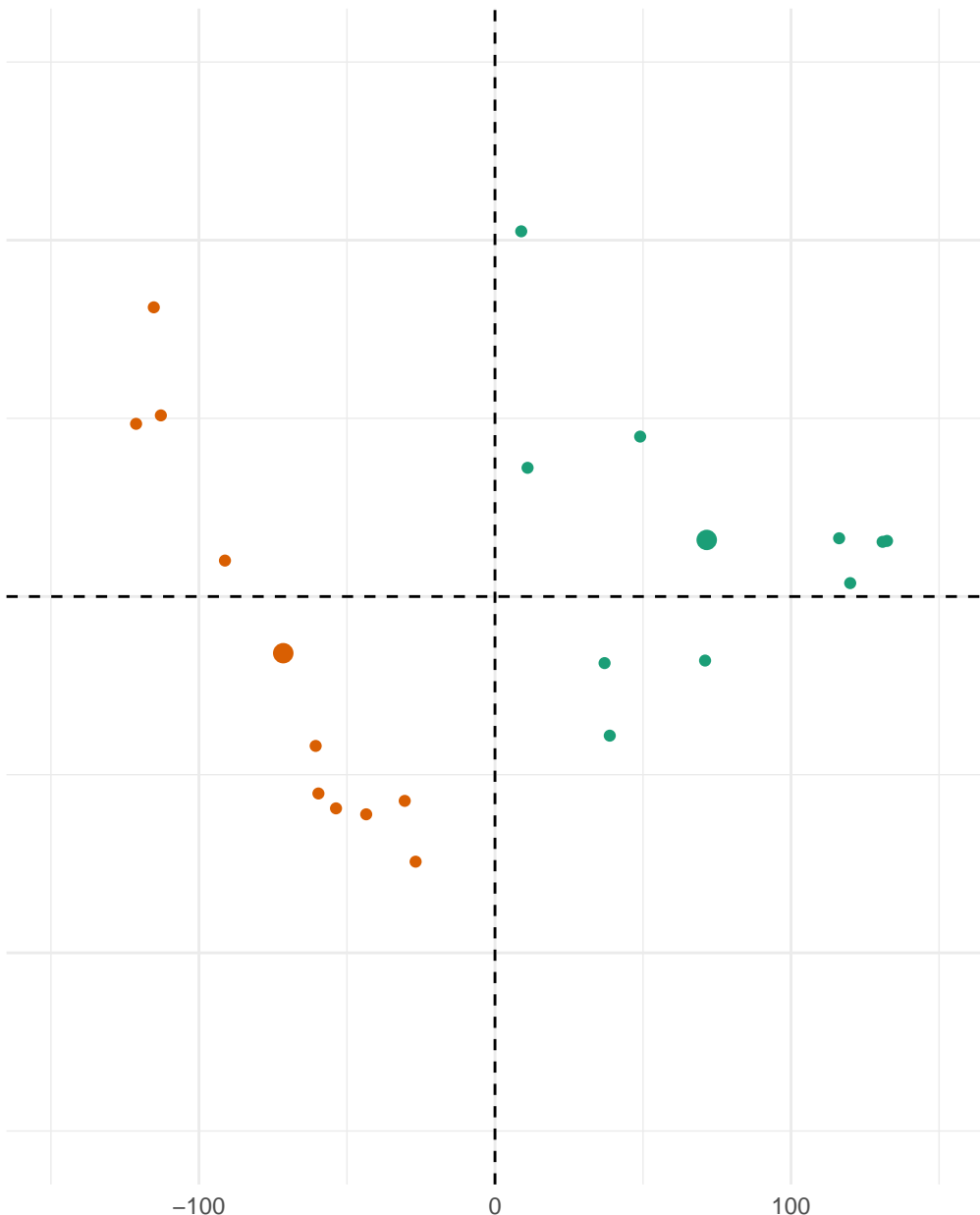

Supplement: Supplementary file 1 [file microorganisms-11-02694-s001.zip › microorganisms-2641477-supplementary/philr_pca_fitted_04.pdf]

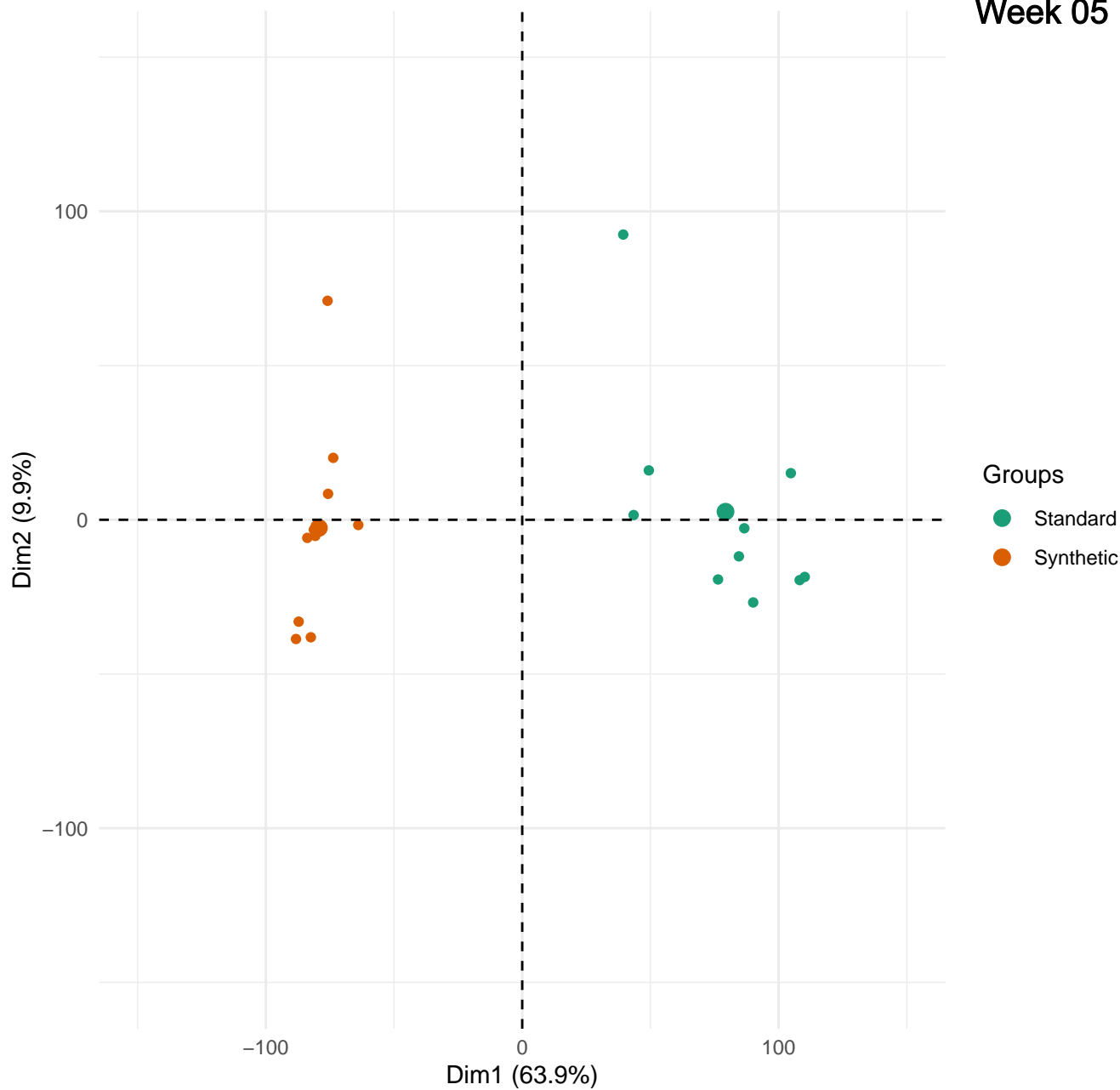

Supplement: Supplementary file 1 [file microorganisms-11-02694-s001.zip › microorganisms-2641477-supplementary/philr_pca_fitted_05.pdf]

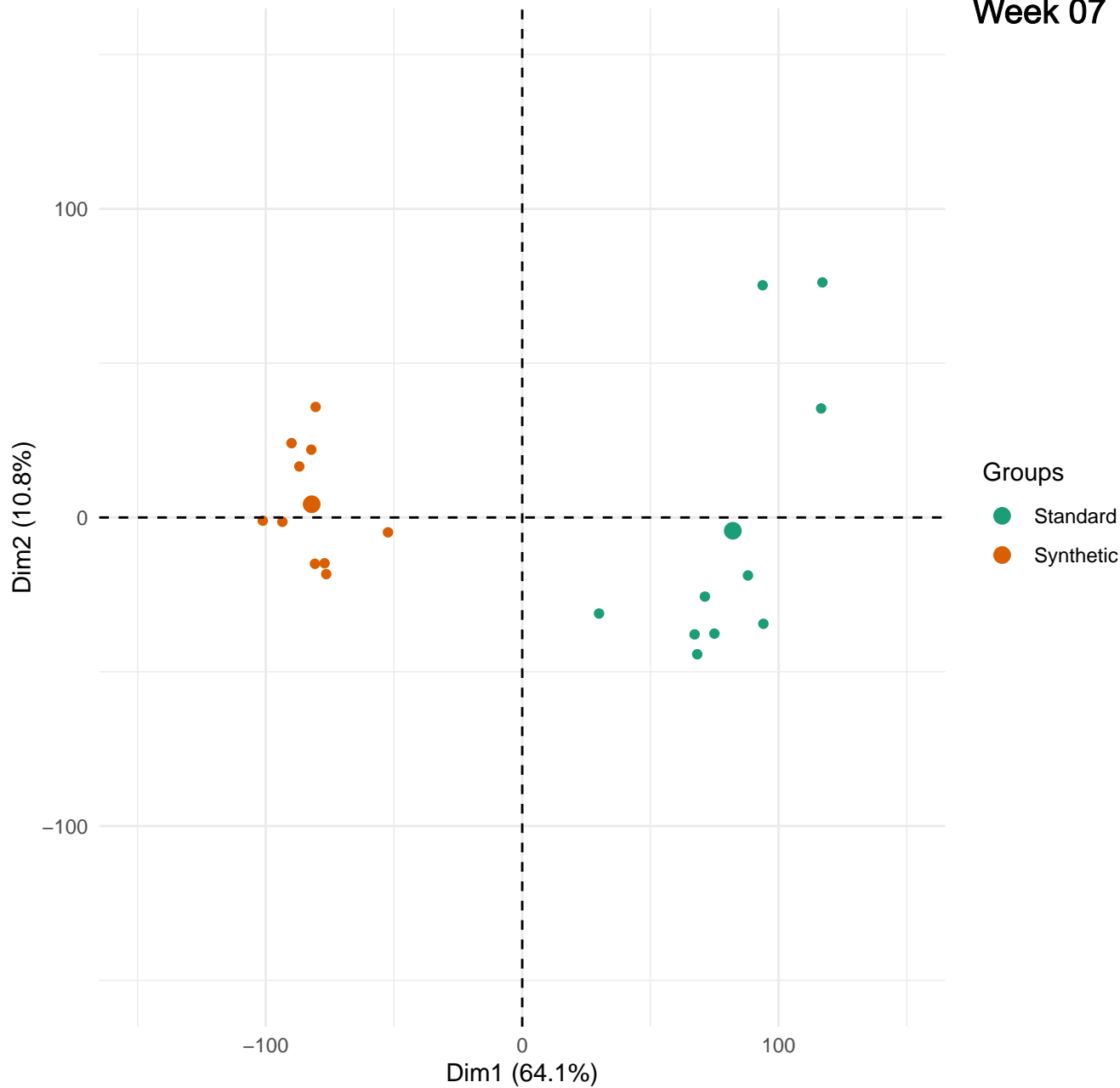

Supplement: Supplementary file 1 [file microorganisms-11-02694-s001.zip › microorganisms-2641477-supplementary/philr_pca_fitted_07.pdf]

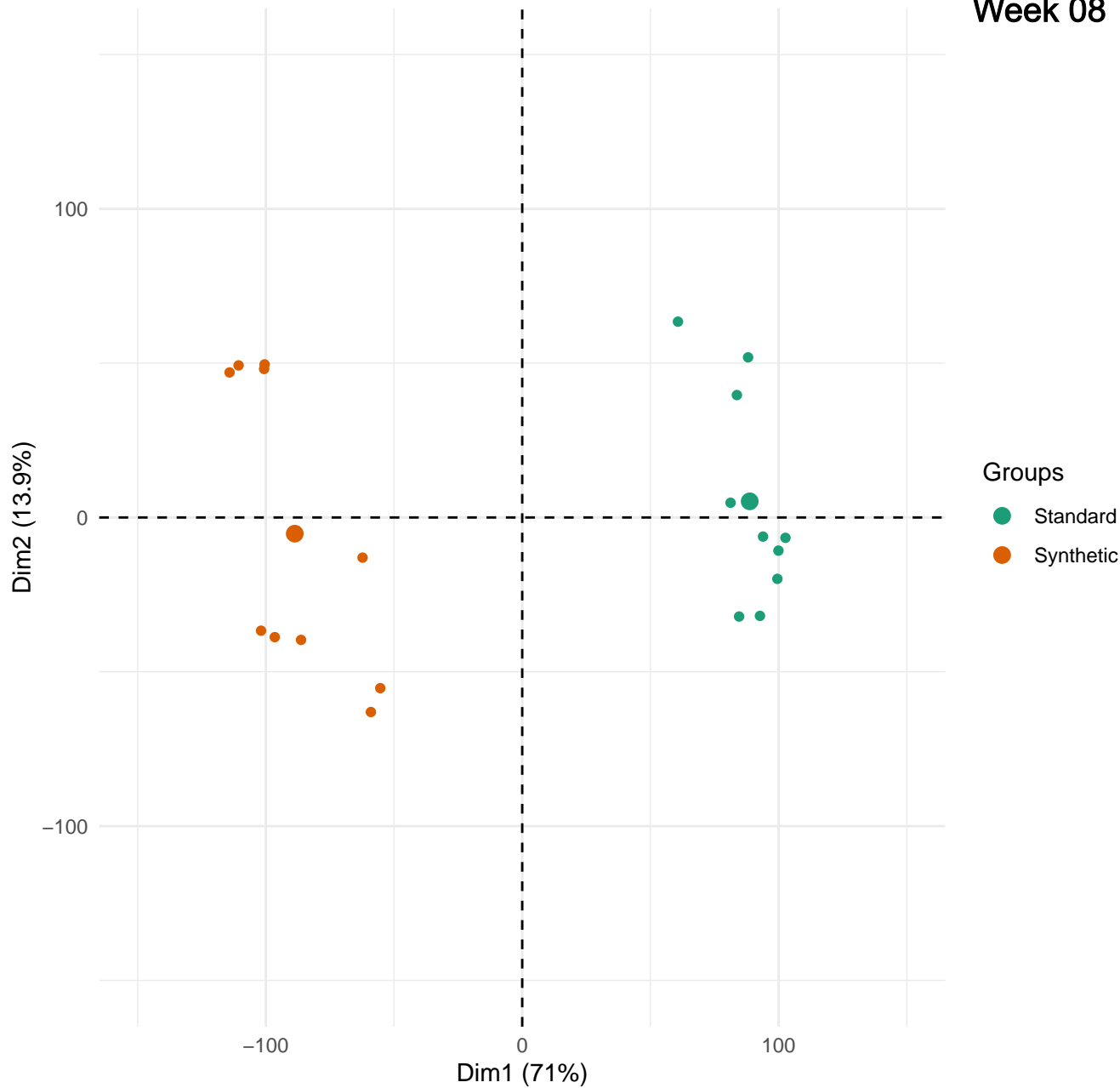

Supplement: Supplementary file 1 [file microorganisms-11-02694-s001.zip › microorganisms-2641477-supplementary/philr_pca_fitted_08.pdf]

Dim2 (7.5%)

Dim1 (80.2%)

Groups

- Standard
- Synthetic

100

0

-100

-100

0

100

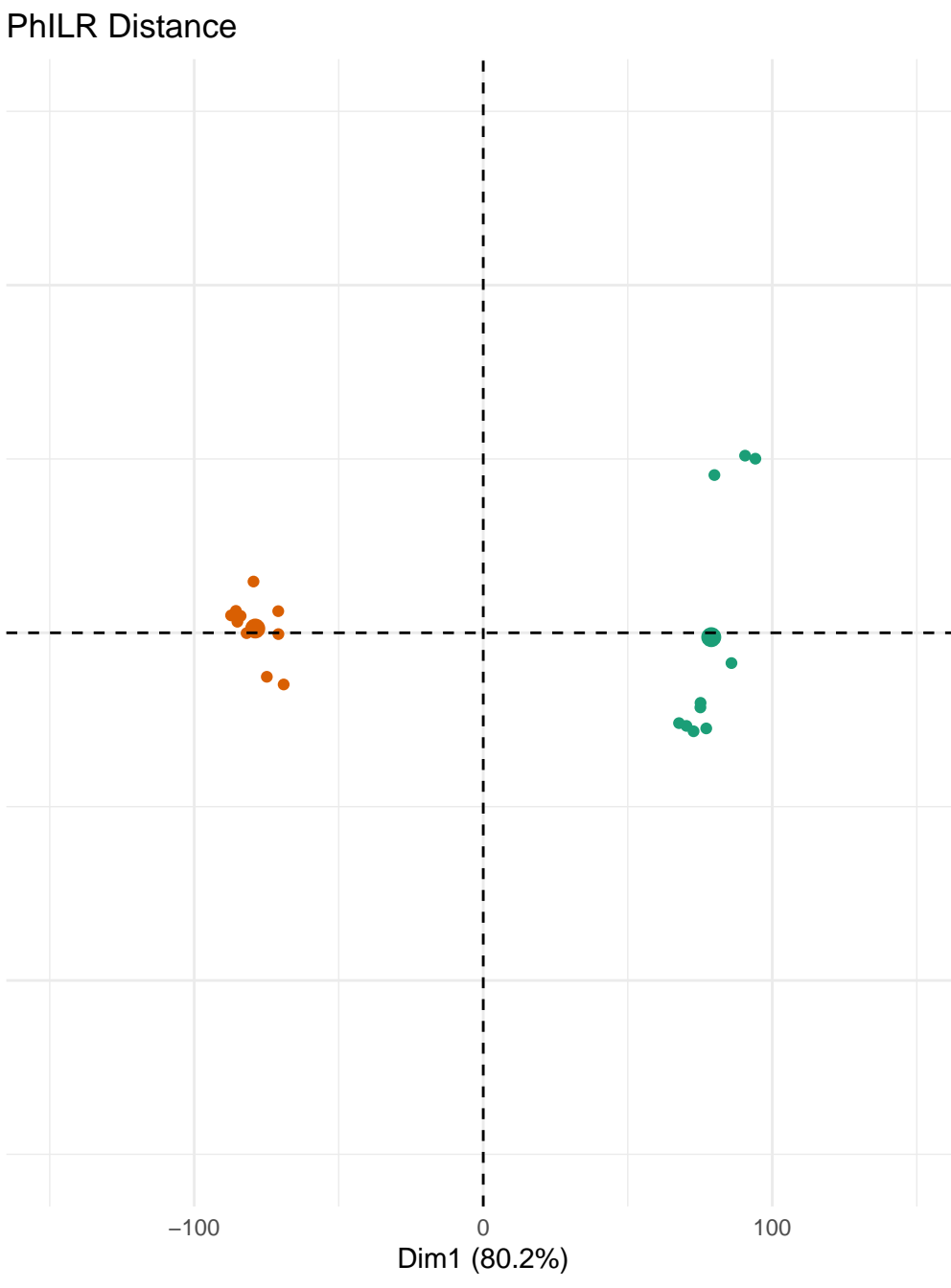

Supplement: Supplementary file 1 [file microorganisms-11-02694-s001.zip › microorganisms-2641477-supplementary/philr_pca_fitted_09.pdf]

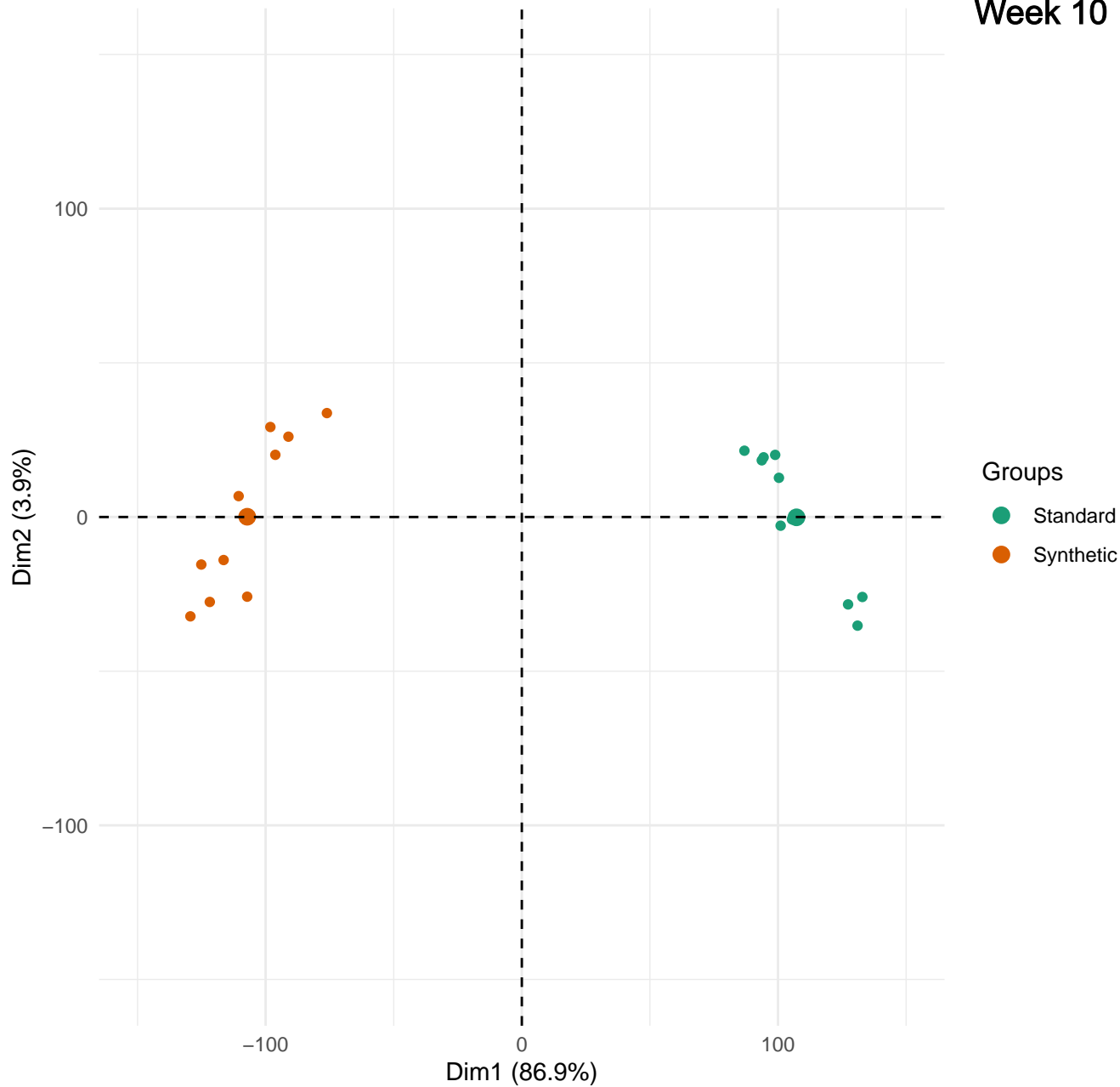

Supplement: Supplementary file 1 [file microorganisms-11-02694-s001.zip › microorganisms-2641477-supplementary/philr_pca_fitted_10.pdf]

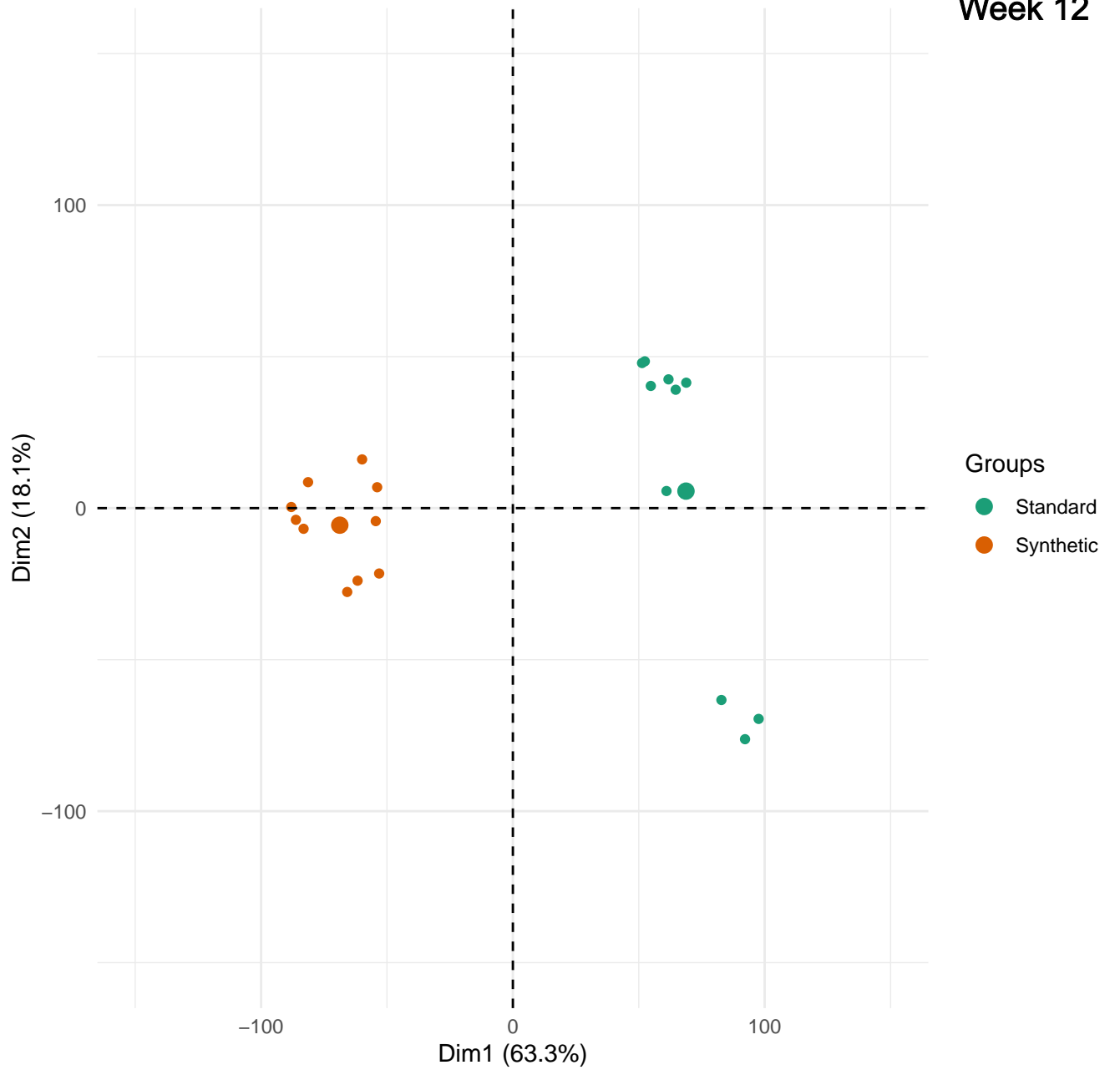

Supplement: Supplementary file 1 [file microorganisms-11-02694-s001.zip › microorganisms-2641477-supplementary/philr_pca_fitted_12.pdf]

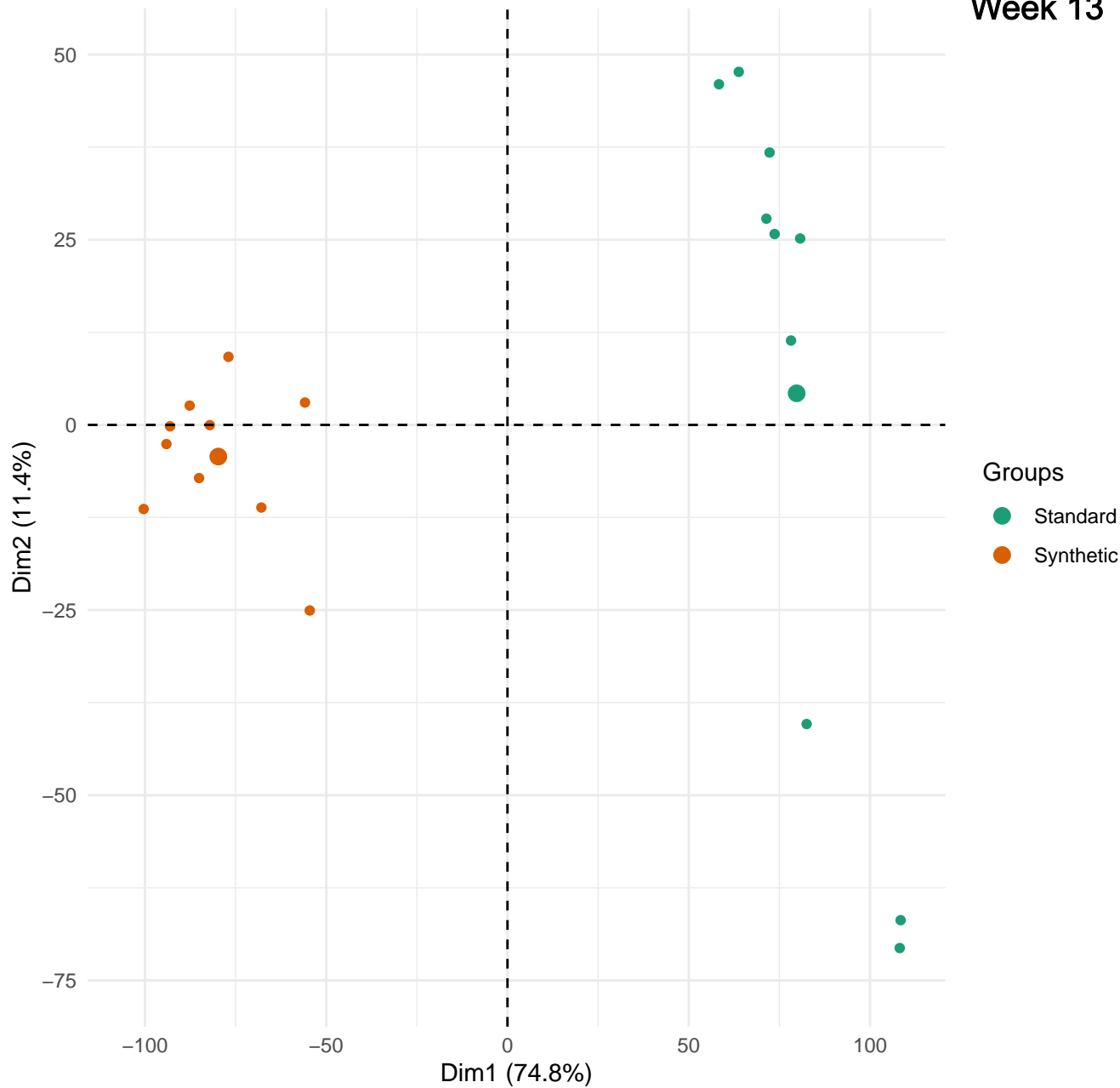

Supplement: Supplementary file 1 [file microorganisms-11-02694-s001.zip › microorganisms-2641477-supplementary/philr_pca_fitted_13.pdf]

PhILR Distance

Week 0

Dim2 (26.6%)

Dim1 (47.2%)

Groups

- Standard
- Synthetic

100

0

-100

-100

0

100

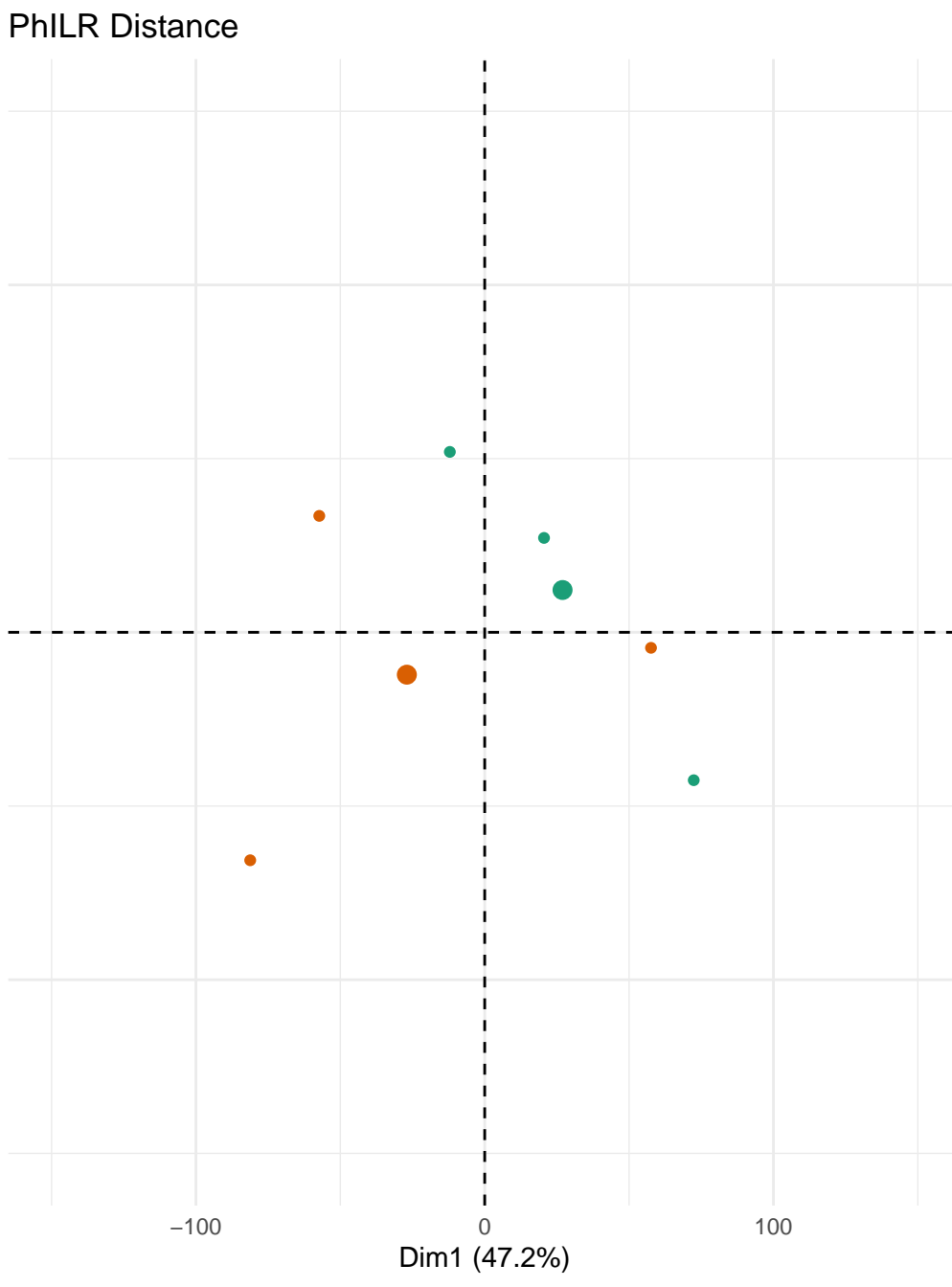

Supplement: Supplementary file 1 [file microorganisms-11-02694-s001.zip › microorganisms-2641477-supplementary/philr_pca_fitted_base.pdf]

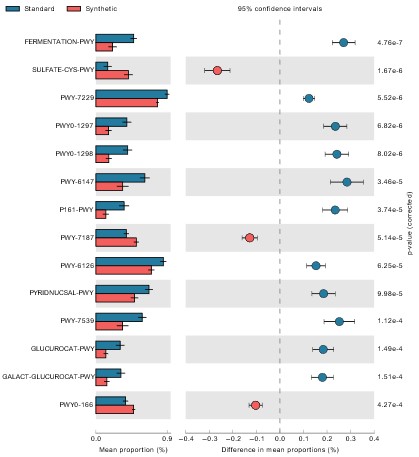

Supplement: Supplementary file 1 [file microorganisms-11-02694-s001.zip › microorganisms-2641477-supplementary/Supplemental 15.jpg]
